# Predicting Late Winter Dissolved Oxygen Levels in Arctic Lakes Using Morphology and Landscape Metrics

**DOI:** 10.1007/s00267-015-0622-x

**Published:** 2015-10-14

**Authors:** Jason C. Leppi, Christopher D. Arp, Matthew S. Whitman

**Affiliations:** The Wilderness Society, 705 Christensen Dr., Anchorage, AK 99501 USA; Water and Environmental Research Center, University of Alaska Fairbanks, 306 Tanana Loop, Fairbanks, AK 99775 USA; Bureau of Land Management, Arctic Field Office, 1150 University Avenue, Fairbanks, AK 99709 USA

**Keywords:** Dissolved oxygen, Overwintering fish habitat, National Petroleum Reserve-Alaska, Arctic lakes, North Slope

## Abstract

Overwintering habitat for Arctic freshwater fish is essential, such that understanding the distribution of winter habitat quality at the landscape-scale is warranted. Adequate dissolved oxygen (DO) is a major factor limiting habitat quality in the Arctic region where ice cover can persist for 8 months each year. Here we use a mixed-effect model developed from 20 lakes across northern Alaska to assess which morphology and landscape attributes can be used to predict regional overwintering habitat quality. Across all lakes, we found that the majority of the variations in late winter DO can be explained by lake depth and littoral area. In shallow lakes (<4 m), we found evidence that additional variables such as elevation, lake area, ice cover duration, and snow depth were associated with DO regimes. Low DO regimes were most typical of shallow lakes with large littoral areas and lakes that had high DO regimes often were lakes with limited littoral areas and deeper water. Our analysis identifies metrics that relate to late winter DO regimes in Arctic lakes that can aid managers in understanding which lakes will likely provide optimum DO for overwintering habitat. Conversely, lakes which predicted to have marginal winter DO levels may be vulnerable to disturbances that could lower DO below critical thresholds to support sensitive fish. In regions where lakes are also used by humans for industrial winter water supply, such as ice-road construction for oil and gas development, these findings will be vital for the management of resources and protection of Arctic fish.

## Introduction

Lakes in the Arctic cover up to 40 % of the landscape (Grosse et al. 2012) and provide important aquatic habitat including overwintering habitat for numerous Arctic fish (West et al. 1992; Morris 2003; Morris et al. 2006; Millar et al. 2013). Long cold winters that are characteristic of the Arctic limit lake occupancy by fish (Haynes et al. 2014) and necessitate that fish develop life history strategies to access suitable overwintering habitat (Morris et al. 2006; Moulton et al. 2007; Brown et al. 2014). In addition to rivers of sufficient depths, deeper lakes that do not freeze to the bed (i.e., floating-ice lakes) can provide important winter habitat for fish. However, these locations also serve as water sources for oil and gas development within the region (Jones et al. 2009, 2013). Limited roads and the sensitivity of permafrost peatland tundra to disturbance necessitate that the majority of travel for exploration and some developments be conducted in the winter using temporary ice roads. To create ice roads, water is either pumped from lakes or ice aggregate is extracted from lake ice during the winter months. Lakes provide a major source of water for ice-road construction, as well as operations for drilling pads and domestic use in camps on the North Slope (Adams 1978; Hinzman et al. 2005). As exploration and development expands, it is likely that water extraction from overwintering fish habitat will increase. If enough highly oxygenated water is removed from a lake, it has the potential to reduce dissolved oxygen (DO) concentrations and impact sensitive fish species and aquatic biota (Cott et al. 2008b, 2015). Therefore, understanding how landscape factors relate to DO in Arctic lakes will inform managers as to which lakes potentially serve as high-quality overwintering habitat and the sensitivity to disturbance from winter water extraction.

The history of modeling DO has been extensive over time and researchers have used both mechanistic (Livingstone and Imboden 1996; White et al. 2008) along with deterministic models (Mathias and Barica 1980; Babin and Prepas 1985; Chambers et al. 2008; Akkoyunlu et al. 2011) to explain DO patterns in lakes. Winter DO budgets are a function of available oxygen in the fall, the duration of ice cover, and the sum of the processes that produce or consume oxygen during the winter, but it is often difficult and expensive to measure these variables. Mechanistic models have been a useful tool to simulate this process at a lake level, but models require more input information than deterministic models to generate accurate results making it difficult to predict habitat quality of lakes in remote locations.

Understanding the variables that influence winter DO has drawn some attention over time due to the sudden die-off of fish largely in the upper Midwestern U.S. and Canada, but early attempts to correlate morphometric variables with a lake’s potential to experience winter anoxic conditions were largely inconclusive (Schindler 1971; Welch 1976; Barica and Mathias 1979). However, early research identified the inverse relationship between mean lake depth and DO depletion (Mathias and Barica 1980). The importance of sediment area to lake volume along lake trophic state was also found to explain a large amount of the variation in winter oxygen depletion rates (Mathias and Barica 1980). More recent limnological investigations have shown that additional variables besides the bathymetry also influence winter DO patterns. For example, percent littoral area surrounding a lake in combination with macrophytes biomass influences biological oxygen demand through winter decomposition (Meding and Jackson 2003). Dissolved organic carbon concentrations have been shown to influence DO, but due to challenging collection logistics these measurements are sparse in northern regions (Clilverd et al. 2009). While much of the previous research has focused on physical variables associated with DO patterns, the occurrence and activity of microorganisms under the ice through aerobic respiration, lithotrophic metabolism, and abiotic oxidation processes (Bertilsson et al. 2013) likely have a significant influence on DO.

Adequate under-ice DO is an important water quality parameter that affects fish respiration, reproduction, growth, and survival. Previous research has documented that most freshwater fish require DO levels greater than ca. 6 mg/L (Davis 1975) with lethal levels potentially occurring below 2 mg/l (Doudoroff and Shumway 1970). Numerous studies have documented species-specific sub-lethal effects on fish which include predation vulnerability, changes in community structure, altered swimming behavior and avoidance success, and reduce reproductive success (Kramer 1987; Pollock et al. 2007). In the Arctic, there are certain tolerant fish species such as Alaska blackfish and ninespine stickleback that inhabit shallow lakes that contain hypoxic winter conditions (Haynes et al. 2014). These fish species reside in shallow lakes year round and are important prey resources for larger fish during the summer months (Heim et al. 2014). Little is known about the adaptive capacity of Arctic fish, but research on Alaskan Blackfish suggests that it is possible for fish to develop adaptive mechanisms to survive harsh conditions (Lefevre et al. 2014).

Current regulations to protect fish are based upon general classes of fish DO sensitivity (i.e., non-sensitive, sensitive) present. This approach has demonstrated to be largely effective since the early 2000s (Hinzman et al. 2006; Chambers et al. 2008; Clilverd et al. 2009), but managers should continue to seek a further understanding of winter lake DO dynamics as increased water demand may require adaptive strategies. Management practices allow for annual water withdrawal amounts based upon modeled lake water volume under-ice and fish species knowledge, but do not require a site-specific assessment on each lake’s winter DO budget. This is a future potential concern for managers as oxygen depletion rates are likely regulated by lake mixing and ecosystem metabolism (Meding and Jackson 2003). Research in the Northwest Territories, Canada has documented that water withdrawal of 20 % under-ice volume in small (<0.3 km^2^) lakes affected oxygen concentrations beyond natural fluctuations (Cott et al. 2008a).

Due to difficulties in conducting water quality measurements during winter, DO concentrations have only been measured sporadically across the Arctic Alaska at lakes associated with oil and gas exploration within the National Petroleum Reserve. Existing measurements from similar lakes within close proximity suggest that the variations in DO are related to a variety of local and regional landscape features, but little information is known about how landscape features are associated with winter DO concentrations. In a region where water extraction is anticipated to expand, increased water use needed for exploration and development has the potential to reduce winter DO in lakes already with naturally low levels.

The goal of this research was to investigate winter DO patterns in Arctic lakes and identify attributes associated with winter DO regimes. DO is an important component of overwintering fish habitat quality and understanding how lake and landscape attributes relate to DO will improve future management of water resources. Our specific objectives were to (1) summarize late winter DO patterns at lakes across the North Slope of Alaska and (2) explore the relationship between landscape and lake morphology metrics and late winter DO. Our research is unique in that we used lake measurements collected as part of the landscape-scale lake monitoring program (CALON), which focuses on lakes from the Brooks Range to the Arctic Coastal Plain of northern Alaska. We use geospatial data to generate a suite of landscape variables thought to correlate with winter DO levels to develop a winter DO statistical model that can provide insights for other parts of the Arctic. This data provide an original perspective on the relationships between winter DO and lake characteristics across a landscape cross section of Arctic North Slope lakes.

## Materials and Methods

### Study Area

**Fig. 1:**
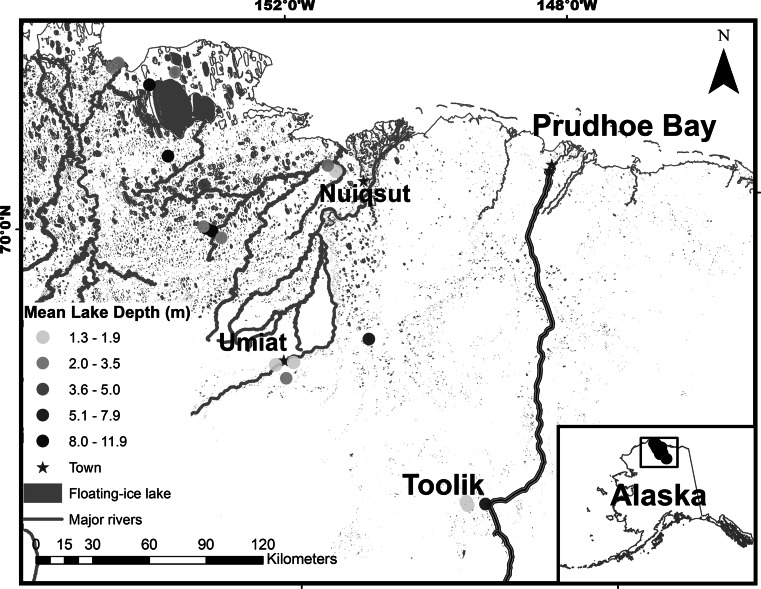
Map of the study area showing the locations of the lakes used in the analysis (*dots*) along with mean lake depth (*color of dots*), major streams (*thin gray lines*), and lakes with winter liquid water (*gray shaded polygons*) as well as the relative position of the study area in northern Alaska (Color figure online)

**Fig. 2:**
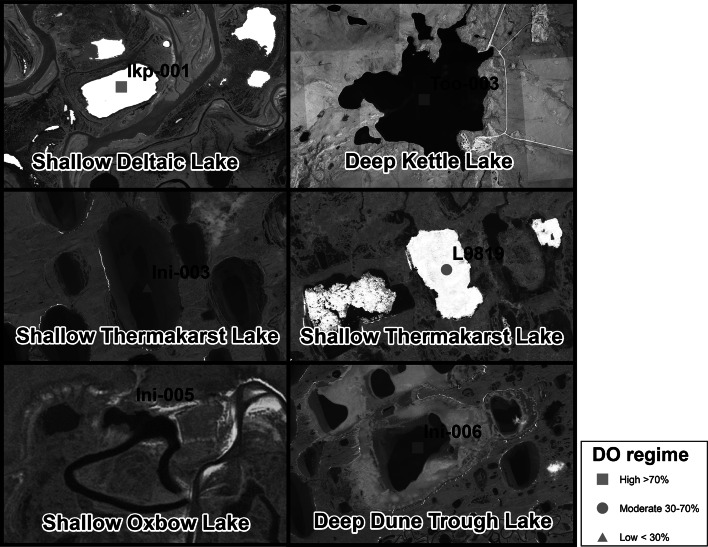
DO regimes and lake images from six example locations within the study area. *Each panel* shows the lake boundary, the DO regime, and the proximity to other water bodies

The study area is located in northern Alaska across an elevational gradient from the foothills of the Brooks Range north to the Beaufort Sea coast, bounded by the Ikpikpuk River on the west and the Dalton highway on the east (Fig. 1). We organized data from all available lakes within the study area that contained late winter (March 23rd–April 23rd) measurements, constituting 20 lakes (Table 1). The study area is around 39,000 km^2^ and encompasses a diverse physiographic and climatic region. Within the area are glaciated foothills with kettle lakes, vegetated eolian sand dunes with deep depression lakes, and coastal plain tundra with shallow thermokarst lakes (Fig. 2). Lake size and depth vary greatly within the study area, but most lakes are shallow (<6 m, range 1.3–18.1 m) with low to moderate levels of productivity (i.e., oligotrophic, mesotrophic). Ice typically forms in October and historically grows up to 2 m thick by late winter. However, a thinning trend documented over the last 30 years suggests maximum ice thickness have declined by 15 cm/decade and now are often less than 1.5 m thick by late April (Arp et al. 2012). Ice-out timing varies widely among lakes depending on depth, surface area, and hydrologic connectivity such that typical breakup dates range from early June to late July (Arp et al. 2013). Lakes within the study area could potentially provide overwintering habitat for the most prevalent fish species such as Arctic grayling (*Thymallus arcticus*), broad whitefish (*Coregonus nasus*), least cisco C*. sardinella*), Alaska blackfish (*Dallia pectoralis*), and ninespine stickleback (*Pungitius pungitius*) (Morris 2003; Morris et al. 2006; Moulton 2005; Moulton et al. 2007, 2010) as well as several other less common species.

**Table 1:** Lake coordinates, morphology, and landscape metrics for all lakes used in the analysis

Lake name	Latitude (DD)	Longitude (DD)	Lake depth (m)	Area (ha)	Elevation (m)	Lake drainage position	Littoral (%)
Ikp-001	70.790	−154.451	3.5	68.68	0.5	2	37
Ini-001	69.996	−153.071	14.0	66.41	40.6	4	39
Ini-003	69.964	−152.949	2.3	417.27	46.9	2	43
Ini-004	70.011	−153.154	11.5	172.46	35.5	3	59
Ini-005	70.019	−153.188	2.6	4.89	38.9	1	86
Ini-006	70.219	−153.172	5.4	361.75	33.5	0	20
L7916	70.299	−151.463	2.5	166.05	0.5	5	27
Lake Helen	70.361	−153.668	11.6	459.83	25.0	4	46
Tes-001	70.766	−153.563	2.5	979.90	3.8	4	6
Too-001	70.706	−153.924	18.1	23.54	698.9	2	16
Too-003	68.633	−149.605	8.9	144.91	719.3	5	7
Too-005	68.649	−149.848	1.7	18.52	609.6	5	21
Umi-004	69.455	−151.008	6.6	63.22	42.7	1	2
Umi-005	69.347	−152.253	2.8	3.02	83.8	2	72
Umi-006	69.281	−152.121	2.4	9.22	164.5	2	21
Ikp-003	70.812	−154.360	3.1	11.22	0.6	1	100
Umi-003	69.356	−152.018	1.6	11.60	81.7	2	26
L9819	70.270	−151.355	1.9	100.51	6.8	4	6
MC7916	70.299	−151.463	2.4	166.05	0.5	5	27
Too-002	68.632	−149.832	1.3	13.74	624.8	3	18

*Lake depth* estimated maximum depth of lake where measurement was taken, *Area* surface area (ha) of the lake using radar imagery, *Elevation* elevation of lake (m), *LDP* lake drainage position rank (values; *0* no connection–*5* highly connected), *Littoral* an estimate of percent lake littoral area (area >1.6 m deep) using radar imagery

### Data

We collected winter DO measurements from 2012 to 2014 in coordination with the CALON network (http://www.arcticlakes.org/). During each sampling period, ice thickness, lake depth, water temperature, and DO measurements were taken from one location drilled through the lake ice estimated to be the deepest part of the lake. Water temperature and DO measurements were recorded at several locations throughout the water column. Within the study area, we also measured daily DO for a subset of lakes (*n* = 5) across the winter season. We used ARCGIS 10.1 to further generate additional metrics for each lake within the study area. Lake morphology metrics (e.g., area, littoral area) were calculated using a shapefile generated from 2009 synthetic aperture radar (SAR) imagery (Grunblatt and Atwood 2014). We included surface area and elevation as a covariate because these variables are thought to influence the timing of lake ice cover formation and thawing, mixing and diffusion processes which likely affect the DO regime. Littoral area was defined as the surface area of each lake that is less than about 1.6 m in depth. This was estimated using the 2009 SAR imagery and corresponds to the area within each lake that freezes solid in the winter. We hypothesize that this is an important factor affecting Arctic lake DO budgets due to the abundance of aquatic macrophytes that are typically found in this zone and the potential of decaying macrophytes to consume large amounts of oxygen (Meding and Jackson 2003). To quantify the effect of lake position within the drainage and seasonal connection, we assigned each lake a drainage position rank (0 = no connection; 1 = ephemeral outlet; 2 = ephemeral inlet and outlet; 3 = perennial outlet; 4 = ephemeral inlet and perennial outlet; 5 = perennial inlet and outlet) based upon the lake seasonal connection to a stream. We expect that the position of the lake and the seasonal stream connection to influence nutrient transport, which may be associated with lake productivity and inversely related to winter DO availability (Martin and Soranno 2006). Additionally, we explore two dynamic variables, ice cover duration and snow depth, that change annually and are known to influence under-ice oxygen production and consumption.

### Statistical Analysis

**Fig. 3:**
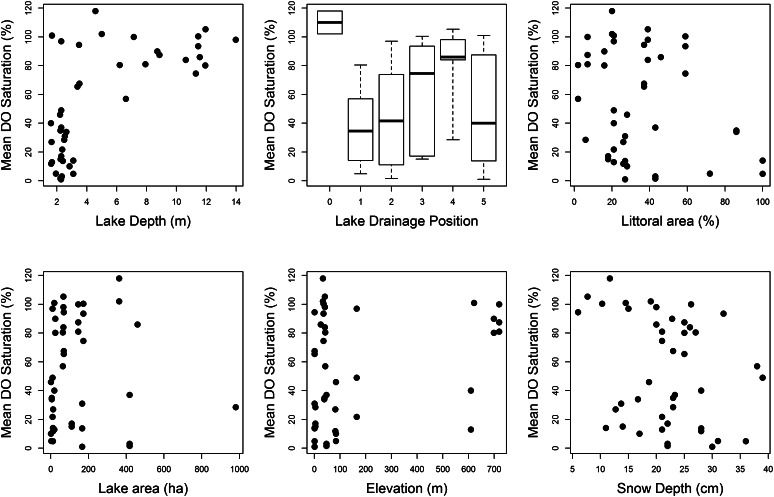
Mean DO saturation percent at all lakes verses landscape and lake morphology metrics. Each *dot* represents a recorded value of Mean DO saturation at a lake and the associated landscape or lake morphology variable

Due to the structure of our data, we chose to use a mixed-effect modeling approach to account for the stochastic nature of the data collected at different sites across space and time. Our analysis was conducted in R statistical platform (R Development Core Team 2014). Exploratory analysis was conducted by visually screening the data for outliers and comparing the relationship between covariates (Fig. 3). To test for multicollinearity, we calculated the variance inflation factors (VIF and GVIF) using corvif function within the AED package (Zuur et al. 2009) and if preliminary covariates had VIFs greater than four they were eliminated as explanatory variables used to build model candidate sets. Temporal correlation was assessed for DO values using visual- as well as calculation-based methods within the stats package. The data analyzed here were complicated by the fact that measurements were taken at different days and depths in lakes. To capture the time period when ice thickness was greatest and DO values are lowest, we restricted our analysis to late winter (March 24th–April 23rd). In order to focus our analysis in areas that are utilized by fish, we average all DO measurements within the top 85 % of the lake and excluded measurements near the bottom of the lake that are typically anoxic all winter. We then used a linear mixed-effect modeling approach where we allowed intercept variance to change (random effect) by lake to account for the similarity of variances within lakes and differences in variances between lakes.

Using landscape and lake morphology variables hypothesized to influence winter DO, we tested all combinations of the following fixed parameters: lake depth (m), lake area (m^2^), lake littoral area (%), lake drainage position (LDP), and elevation (m) for all our sites. The advantage of testing all combination of ecologically relevant parameters is that we can determine the relative importance of parameters (Burnham and Anderson 2002, chap 4). Next, in order to understand if fluctuating site attributes (dynamic variables) influence winter DO, we tested an additional model set that included the parameters ice cover duration and snow depth where data were available at sites. Within our model selection process, we also explored the same set of parameters on DO for our shallow lakes (depth <4 m). All candidate models were fitted using maximum likelihood estimation and compared using second-order Akaike information criterion (AICc) to identify the most parsimonious model. Candidate models with delta AICc’s less than four were selected as competing models (Table 3). All models that had nested values within two delta AICc of the top model were considered to have uninformative parameters, excluded from the final model set (Table 3) and assumed not to be ecologically important (Arnold 2010). The final model sets was then rerun using restricted maximum likelihood estimation and residuals were assessed visually for heteroscedasticity and normality. Model accuracy was assessed by examining the marginal and conditional *R*^2^ values (Nakagawa and Schielzeth 2013).

## Results

### Dissolved Oxygen Patterns

**Fig. 4:**
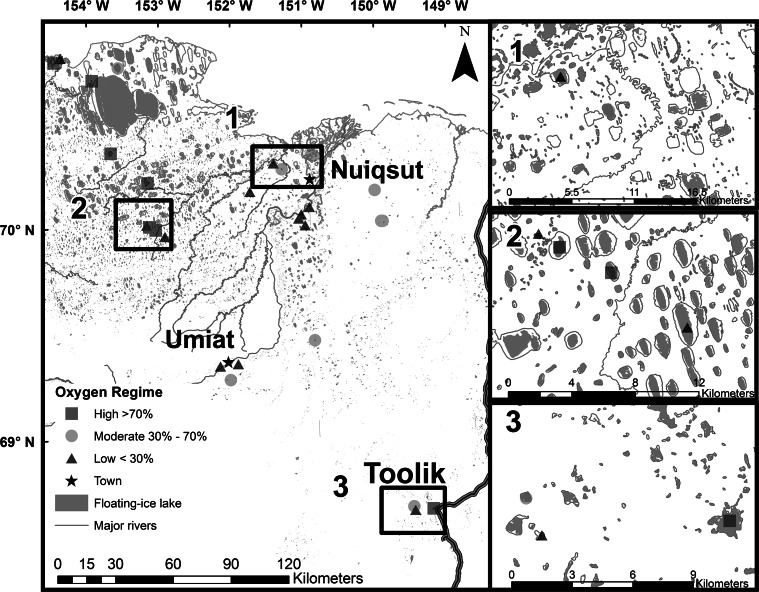
DO regimes; low (DO <30 %), Moderate (30 % ≤ DO ≤ 70 %), High (DO ≥70 %) of the lakes used in the analysis along with *inset* maps for areas with dense clusters of lakes. *Each symbol* represents one of the three DO regimes, major streams are shown as *thin gray lines,* and lakes with winter liquid water are represented as *shaded gray polygons*

**Fig. 5:**
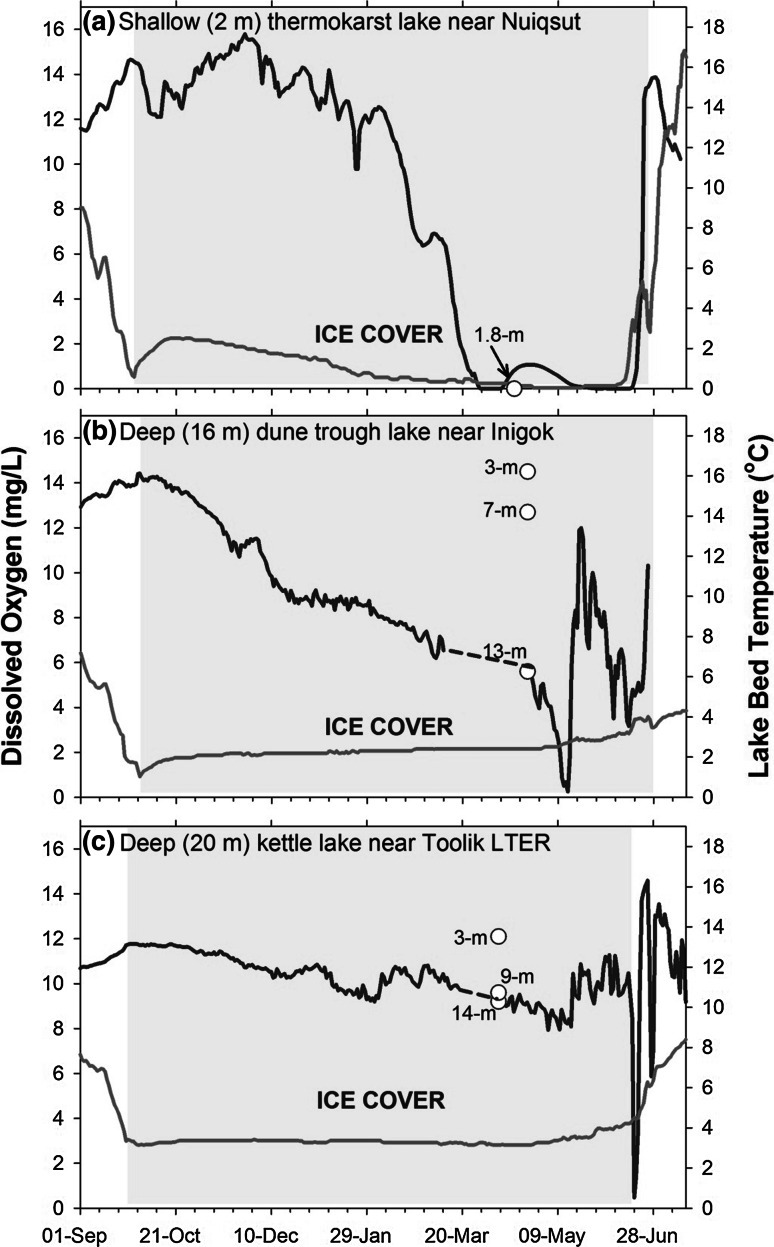
Example of a typically winter DO recession curve (*blue line*) and lake bed temperature (*gray line*) for three common lake types (thermokarst, dune trough, kettle lake). The *light blue shaded area* represents the winter ice cover period (Color figure online)

The Arctic lakes included in the analysis encompassed sites across the North Slope with a broad range of attributes (Table 1) and DO regimes (Table 2). Observed mean DO saturation at sites ranged from c.a. 9–103 % with a mean of 49 % (Table 2). Lakes were classified as low (DO <30 %), moderate (30 % ≤ DO ≤ 70 %), and high (DO >70 %) based upon mean DO concentration within the upper 85 % of the lake column (Table 2). Water temperature ranged from 0 to 2. 6 °C during measurements and therefore the DO classes contain the following mg/l of oxygen; low (0–4 mg/L), moderate (4–10 mg/L), and high (10–15 mg/L). Of the total 20 sites, eight had low DO with a mean of around 15 % (range 9–20 %), five had moderate DO with a mean around 50 % (range 32–69 %), and seven had high DO with a mean of about 89 % (range 75–103 %). The pattern of DO regimes (Fig. 4) shows that a variety of regimes exist across the study area and in several locations DO ranged from low to high in adjacent lakes suggesting no obvious spatial pattern. Daily DO measurements at three common types of Arctic lakes show that DO is greatest in the fall when lake ice begins to form, but decreases in unique ways at each lake over the winter (Fig. 5). Generally, the daily DO measurements reveal that the largest most rapid decline occurs in the shallow thermokarst lake, a gradual moderate decline occurs in the deep dune trough lake, and a minimal decline in the deep kettle lake (Fig. 5). Depletion rates at sites (*n* = 5) does appear to be associated with lake depth, but minimal annual variation exists at measured sites. All the sites show numerous small increases in DO over the course of the winter and a large increase in DO at ice breakup.

**Table 2:** Data source and descriptive statistics for lakes used in the analysis

Lake	Data source	Year(s)	Measurements (#)	Mean DO (%)	SD	Oxygen regime
Ikp-001	CALON	2012, 2013, 2014	5	75.8	14.5	H
Ini-001	CALON	2012, 2013, 2014	7	95.5	12.3	H
Ini-003	CALON	2012, 2013, 2014	4	11.2	17.3	L
Ini-004	CALON	2012, 2013, 2014	6	91.0	12.8	H
Ini-005	CALON	2012, 2013, 2014	4	18.8	18.2	L
Ini-006	CALON	2012, 2013, 2014	7	103.1	11.7	H
L7916	CALON	2012, 2013, 2015	4	16.0	21.2	L
Lake Helen	CALON	2012	3	86.0	36.7	H
Tes-001	CALON	2012, 2013, 2014	3	40.8	14.5	M
Too-001	CALON	2012, 2013, 2014	4	83.0	8.9	H
Too-003	CALON	2012, 2013, 2014	3	89.8	9.0	H
Too-005	CALON	2012, 2013, 2014	3	51.3	45.1	M
Umi-004	CALON	2012, 2014	3	68.8	18.0	M
Umi-005	CALON	2012, 2013, 2014	4	17.8	19.5	L
Umi-006	CALON	2012, 2013, 2014	3	55.9	38.1	M
Ikp-003	CALON	2013, 2014	2	9.5	6.4	L
Umi-003	CALON	2013, 2014	2	19.4	10.7	L
L9819	CALON	2014	3	32.5	2.1	M
MC7916	CALON	2014	3	13.8	0.5	L
Too-002	CALON	2014	1	17.0	NA	L

*Mean DO (%)* mean DO value for late winter (March 20–April 30) within the top 85 % of the lake column, *SD* standard deviation, *Oxygen regime* oxygen regime (values; *L* low, *M* moderate, *H* high)

### Dissolved Oxygen Model

To explore the association between landscape and lake morphology variables and winter DO, we tested two models types: (1) static model with fixed landscape and lake morphology variables and (2) a dynamic model with both static and dynamic variables that change annually. We explored our fixed and dynamic models for all lakes as well as a subset of shallow lakes (depth <4 m). For our static model, we found that lake depth (m) and littoral area (%) were the most important variables to predict DO for all lakes (Table 3). The model had a moderate fit (m*R*^2^ = 0.51, c*R*^2^ = 0.64) and the relationship identifies that the fixed parameter explains 51 % of the variation in DO, while the combined fixed and random effects explain 64 % of the variation (Table 3). For shallow lakes within the static model, our results suggest that in addition to lake depth, the covariate lake elevation has some relative importance, but the model had a weak fit. Our dynamic model results show that lake depth and ice cover duration to be the most important variables within the model set and it had a moderate fit (m*R*^2^ = 0.52, c*R*^2^ = 0.84) with a larger amount of variation explained by the combined fixed and random effects. Restricting the dynamic model selection for shallow lakes, our results suggest that additional variables, such as snow depth, elevation, and lake area, influence DO, but results should be interpreted with caution due to the reduced sample size.

**Table 3:** AIC table with estimates of *R* ^2^ for all linear mixed-effect models with <4 delta AIC from top model

Model description		Fixed effects						*n*	df	∆AICc	AICcwt	m*R* ^2^	c*R* ^2^
		Area	Elev	LakeD	Littoral	IceD	Snow						
Landscape 1				6.49	−0.35			43	5	0.00	0.36	0.51	0.64
Landscape 1				6.51				43	4	0.94	0.22	0.45	0.64
Landscape 2			0.09	29.63				27	5	0.00	0.28	0.28	0.28
Landscape 2			0.04					27	4	2.24	0.09	0.08	0.29
Landscape 2					−0.23			27	4	2.85	0.07	0.04	0.27
Landscape 2				10.13				27	4	3.08	0.06	0.04	0.26
Dynamic 1				5.30	−0.56	−1.10		21	6	0.00	0.64	0.64	0.89
Dynamic 1				5.33		−1.05		21	5	1.17	0.36	0.49	0.89
Dynamic 2						−1.11		13	4	0.00	0.34	0.25	0.74
Dynamic 2							−2.33	13	4	2.44	0.10	0.18	0.63
Dynamic 2			0.07					13	4	2.72	0.09	0.21	0.21
Dynamic 2		−7.97E-06						13	4	3.81	0.05	0.14	0.20
Dynamic 2					−0.38			13	4	3.97	0.05	0.12	0.41

Models: *Landscape 1* landscape model with all sites, *Landscape 2* landscape model with only shallow (lake depth <4 m) sites, *Dynamic 1* dynamic model with all sites that have ice on dates and snow depth, *Dynamic 2* dynamic model with shallow sites (lake depth <4 m) that have ice on dates and snow depth. Fixed effects used in the model: *Depth* maximum lake depth, *Area* surface area (m^2^) of the lake using radar imagery, *Littoral* an estimate of percent lake littoral area (area <1.6 m deep) using radar imagery, *LDP* lake drainage position rank (values; *0* no connection–*5* highly connected). Dynamic variables, *IceD* the number of days that the lake was covered in ice, *Snow* snow depth on top of the lake ice. Additional info; *DF* the degrees of freedom in model, *AIC* Akaike information criterion, *∆AIC* change in AIC values, *AICwt* the relative likelihood of a model, *mR* ^*2*^ variation explained by the fixed effects model, *cR* ^*2*^ variation explained by the random effects model

## Discussion

Our results suggest that winter DO levels are associated with lake morphology, landscape position, and certain environmental conditions at each lake, which likely influence whether a lake will experience hypoxic conditions. Lakes with low winter DO levels were typically associated with shallow lakes that had large littoral areas. In lakes where ice measurements exist, the onset and duration of ice cover also appear to negatively influence winter DO. Conversely, lakes with high DO levels were typically deeper lakes, with small littoral areas that had a shorter ice cover period. The effect of lake depth on DO levels was greatest, compared to the rest of our hypothesized factors and explained about 45 % of the variation in DO levels. However, within shallow lakes (lakes <4 m deep), our results suggest that lake elevation, area, and snow depth have some role in influencing the variation in DO between lakes.

Our results corroborate early research by Mathias and Barica (1980) that found that oxygen depletion rates were slower in deeper lakes and the area of sediment–water interface and littoral zone in relation to total volume influenced the winter oxygen depletion. Depth has a dominant role in influencing winter DO due to the inverse correlation between depth and total phosphorus and total carbon loading (Meding and Jackson 2003). Depth also influences DO vertical transport and within deeper lakes the mixing of stratified layers is greatly reduced (Molot et al. 1992). Our results support the concept that deeper lakes are less likely to become hypoxic because they have less relative contact with the lake bed interface, generally have lower productivity, and are thus able to retain higher DO levels compared to shallow lakes.

Lakes with large littoral areas are likely to have reduced winter DO because of increased exposure to the lake sediment interface where microbial processes consume oxygen through decomposition and oxidation (Muller et al. 2012). Greater sediment area also increases the heat accumulation capacity during the open water period and influences thermally driven under-ice mixing which facilitates oxygen consumption (Malm 1999; Kirillin et al. 2012). In addition, littoral areas often contain dense macrophyte coverage which through the processes of decomposition during winter consumes oxygen (Meding and Jackson 2003). Our results provide further support for the influence of lake morphology on DO and provide further evidence that littoral areas influence DO regimes. In our study area, littoral area was the second most important variable, but it is likely that additional environmental variables interact with lake morphology factors to influence a lake DO regime.

Despite the influence that lake morphology has on DO regimes, we also found evidence that ice cover duration influences winter DO. In lakes where data exist, lakes that had longer ice coverage duration also had lower DO. Ice cover duration is a metric that is influenced by ice formation variables such as wind, air temperature, and heat storage (Brown and Duguay 2010). Once ice cover is established, all physical and biological processes are isolated from the atmosphere greatly reducing, photosynthesis, mixing, and gas and heat exchange (Bertilsson et al. 2013). Therefore, lakes with a longer ice cover duration experience isolated conditions with restricted oxygen inputs longer than lakes with later ice formation dates.

Considerable DO variation (ca. 9–75 %) exists for shallow lakes (<4 m), which suggests that additional variables besides lake depth have a significant influence on DO in shallow lakes. We further explored the association between attributes in shallow lakes and found that in lakes shallower than four meters, elevation, lake area, and snow depth also influenced DO. Previous research has shown that ice formation is influenced by both lake area and elevation with high elevation lakes having a longer ice cover period (Livingstone and Adrianb 2009). Lake size is also likely associated ice formation because larger lakes tend to have stronger wind patterns, due to increased fetch which facilitates mixing and delays ice formation (Brown and Duguay 2010). Once ice cover is established, the duration and amount of snow cover on the ice will also influence light transmission and DO by restricting oxygen production by photosynthesis to the upper water column (Bertilsson et al. 2013).

Our results demonstrate the importance of several landscape features in modeling DO levels in Arctic lakes and highlight the large amount of variation that exists for shallow lakes (lakes <4 m). This research identifies that the lake morphometry variables coupled with other landscape attributes are important factors that influence late winter DO levels and the potential quality of overwintering fish habitat. Future research should be conducted in shallow Arctic lakes in other Arctic regions in order to understand the broader applicability of these results. Lakes deeper than 4 m are likely to provide quality overwintering habitat, but considerable variation exists for shallow lakes and further analysis should be conducted to understand the biological and chemical mechanisms behind the differences in DO levels prior to water extraction. Our results can be used as a first step for management of water resources in the Arctic and if coupled with site monitoring it could be an effective method to identify important overwintering fish habitat and effectively manage development in the Arctic.

## References

[CR1] Adams KM (1978) Building and operating ice roads in Canada and Alaska. Department of Indian and Northern Affairs. North of 60 Environmental Studies No. 4. Minister of Supplies and Services Canada

[CR2] Akkoyunlu A, Altun H, Cigizoglu HK (2011). Depth-integrated estimation of dissolved oxygen in a lake. J Environ Eng.

[CR3] Arnold TW (2010). Uninformative parameters and model selection using Akaike’s Information Criterion. J Wildl Manag.

[CR4] Arp CD, Jones BM, Lu Z, Whitman MS (2012). Shifting balance of thermokarst lake ice regimes across the Arctic Coastal Plain of northern Alaska. Geophys Res Lett.

[CR5] Arp CD, Jones BM, Grosse G (2013). Recent lake ice-out phenology within and among lake districts of Alaska, USA. Limnol Oceanogr.

[CR6] Babin J, Prepas EE (1985). Modeling winter oxygen depletion rates in ice-covered temperate zone lakes in Canada. Can J Fish Aquat Sci.

[CR7] Barica J, Mathias JA (1979). Oxygen depletion and winterkill risk in small prairie lakes under extended ice cover. J Fish Res Board Can.

[CR8] Bertilsson S, Burgin A, Cayelan C (2013). The under-ice microbiome of seasonally frozen lakes. Limnol Oceanogr.

[CR9] Brown LC, Duguay CR (2010). The response and role of ice cover in lake-climate interactions. Prog Phys Geogr.

[CR10] Brown R, Loewen M, Tanner T (2014). Overwintering locations, migrations, and fidelity of radio-tagged Dolly Varden in the Hulahula River, Arctic National Wildlife Refuge, 2007–09. ARCTIC.

[CR11] Burnham K, Anderson D (2002). Model selection and multimodel inference: a practical information-theoretic approach.

[CR12] Chambers MK, White DM, Lilly MR, Hinzman LD, Hilton KM, Busey RC (2008). Exploratory analysis of the winter chemistry of five lakes on the North Slope of Alaska. J Am Water Resour Assoc.

[CR13] Clilverd H, White D, Lilly M (2009). Chemical and physical controls of the oxygen regime of ice-covered Arctic lakes and reservoirs. J Am Water Resour Assoc.

[CR14] Cott PA, Sibley PK, Gordon AM, Bodaly RAD, Mills KH, Somers WM, Fillatre GA (2008). Effects of water withdrawal from ice-covered lakes on oxygen, temperature, and fish. J Am Water Resour Assoc.

[CR15] Cott PA, Sibley PK, Somers WM, Lilly MR, Gordon AM (2008). A review of water level fluctuations on aquatic biota with an emphasis on fishes in ice-covered lakes. J Am Water Resour Assoc.

[CR16] Cott PA, Schein A, Hanna BW, Johnston TA, MacDonald DD, Gunn JM (2015). Implications of linear developments on northern fishes. Environ Rev.

[CR17] Davis JC (1975). Minimal dissolved-oxygen requirements for aquatic life with emphasis on Canadian species. J Fish Res Board Can.

[CR18] Doudoroff D, Shumway DL (1970) Dissolved oxygen requirements of freshwater fishes. FAO Fisheries Technical Paper No. 86. Food and Agricultural Organization of the United Nations

[CR19] Grosse G, Jones B, Arp C, Shroder J, Giardino R, Harbor J (2012). Thermokarst lake, drainage and drained basins. Treatise on geomorphology.

[CR50] Grunblatt J, Atwood D (2014) Mapping lakes for winter liquid water availability using SAR on the North Slope of Alaska. Int J Appl Earth Obs Geoinf 27:63–69

[CR20] Haynes T, Rosenberger A, Lindberg M, Whitman M, Schmutz J (2014). Patterns of lake occupancy by fish indicate different adaptations to life in a harsh Arctic environment. Freshw Biol.

[CR21] Heim K, Wipfli M, Whitman S, Seitz A (2014). Body size and condition influence migration timing of juvenile Arctic grayling. Ecol Freshw Fish.

[CR22] Hinzman LD, Bettez ND, Bolton WR (2005). Evidence and implications of recent climate change in northern Alaska and other arctic regions. Clim Change.

[CR23] Hinzman LD, Lilly MR, Kane DL, et al. (2006) Physical and chemical implications of Mid-Winter Pumping of Tundra Lakes—North Slope, Alaska. December 2006, University of Alaska Fairbanks, Water and Environmental Research Center, Report INE/WERC 06.15, Fairbanks

[CR24] Jones BM, Arp CD, Hinkel KM, Beck RA, Schmutz JA, Winston B (2009). Arctic lake physical processes and regimes with implications for winter water availability and management in the National Petroleum Reserve Alaska. Environ Manag.

[CR25] Kirillin G, Lepparanta M, Terzhevik A (2012). Physics of seasonally ice-covered lakes: a review. Aquat Sci.

[CR26] Kramer DL (1987). Dissolved-oxygen and fish behavior. Environ Biol Fishes.

[CR27] Lefevre S, Damsgaard C, Pascale D (2014). Air breathing in the Arctic: influence of temperature, hypoxia, activity and restricted air access on respiratory physiology of Alaska blackfish (*Dallia pectoralis*). J Biol Exp.

[CR28] Livingstone DM, Adrianb R (2009). Modeling the duration of intermittent ice cover on a lake for climate-change studies. Limnol Oceanogr.

[CR29] Livingstone DM, Imboden DM (1996). The prediction of hypolimnetic oxygen profiles: a plea for a deductive approach. Can J Fish Aquat Sci.

[CR30] Malm J (1999). Some properties of currents and mixing in a shallow ice-covered lake. Water Resour Res.

[CR31] Martin SL, Soranno PA (2006). Lake landscape position: relationships to hydrologic connectivity and landscape features. Limnol Oceanogr.

[CR32] Mathias JA, Barica J (1980). Factors controlling oxygen depletion in ice-covered lakes. Can J Fish Aquat Sci.

[CR33] Meding ME, Jackson LJ (2003). Biotic, chemical, and morphometric factors contributing to winter anoxia in prairie lakes. Limnol Oceanogr.

[CR34] Millar N, Harris L, Howland K (2013). Seasonal migration of broad whitefish (*Coregonus nasus* (Pallas)) in an Arctic lake. Adv Limnol.

[CR35] Molot LA, Dillon PJ, Clark BJ, Neary BP (1992). Predicating end-of-summer oxygen profiles in stratified lakes. Can J Fish Aquat Sci.

[CR36] Morris W (2003) Seasonal movements and habitat use of Arctic grayling (*Thymallus arcticus)*, burbot (*Lota lota*), and broad whitefish (*Coregonus Nasus*) within the Fish Creek drainage of the National Petroleum Reserve-Alaska, 2001-2002. Technical Report No. 03-02. Alaska Department of Natural Resources, Office of Habitat and Management and Permitting

[CR37] Morris W, Moulton L, Bacon J, Rose J, Whitman M (2006) Seasonal movement and habitat use by broad whitefish (*Coregonus nasus*) in the Teshekpuk Lake region of the National Petroleum Reserve-Alaska. Technical Report No. 06-04. Alaska Department of Natural Resources, Office of Habitat and Management and Permitting

[CR38] Moulton L (2005) Baseline surverys of fish habitats in eastern NPR-A, 2004. Final Report for ConocoPhillips and Anadarko Petroleum Corp. 56p

[CR39] Moulton L, Morris W, George C, Bacon J, Rose J, Whitman M (2007) Surveys of fish habitat in the Teshekpuk Lakes region, 2003-2005. Final Report December 2007

[CR40] Moulton L, Whitman M, Morris W, George J, Bacon J, Rose J (2010) Surveys of fish in the Teshekpuk lake region during 2006-2007, with comparisons to previous sampling. Prepared for North Slope Borough Department of Wildlife Management

[CR51] Muller B, Bryant LD, Matzinger A, Wiiest A (2012). Hypolimnetic oxygen depletion in eutrophic lakes. Environ Sci Technol.

[CR41] Nakagawa S, Schielzeth H (2013). A general and simple method for obtaining R^2^ from generalized linear mixed-effects models. Methods Ecol Evol.

[CR42] Pollock MS, Clarke LMJ, Dube MG (2007). The effects of hypoxia on fishes: from ecological relevance to physiological effects. Environ Rev.

[CR43] R Core Team (2014). R: a language and environment for statistical computing. R Foundation for Statistical Computing, Vienna, Austria. ISBN 3-900051-07-0. http://www.R-project.org/

[CR52] Schindler DW (1971). A hypothesis to explain differences and similarites amoung lakes in the Experimental Lakes Area, northwestern Ontario. J Fish Res Board Can.

[CR53] Welch HE, Dillon PJ, Sreedharan A (1976). Factors affecting winter respiration in Ontario lakes. J Fish Res Board Can.

[CR44] West RL, Smith MW, Barber WE, Reynolds JB, Hop H (1992). Autumn migration and overwintering of Arctic grayling in coastal streams of the Arctic National Wildlife Refuge, Alaska. Trans Am Fish Soc.

[CR45] White DM, Clilverd HM, Tidwell AC, Little L, Lilly MR, Chambers M, Reichardt D (2008). A tool for modeling the winter oxygen depletion rate in arctic lakes. J Am Water Resour Assoc.

[CR46] Zuur A, Ieno E, Walker N, Saveliev A, Smith G (2009). Mixed effects model and extensions in ecology with R.

